# Self-Care Behavior and Associated Factors of Nursing Students with Dysmenorrhea: A Structural Equation Model

**DOI:** 10.1155/2023/8820772

**Published:** 2023-11-03

**Authors:** Jinpei Chen, Yajing Duan, Yongai Zhang, Xiaona Zhang, Miao Chu, Yurun Shi, Xinmin Zhang

**Affiliations:** ^1^The Medical School, Yan'an University, Yan'an, China; ^2^The Faculty of Nursing and Rehabilitation, Xi'an Medical University, Xi'an, China

## Abstract

**Objective:**

To assess the factors influencing the self-care behavior among nursing students with dysmenorrhea.

**Background:**

The practice of self-care behavior for dysmenorrhea has gradually attracted immense attention from society; however, thus far, only a few studies have been conducted to predict this behavior and analyze the associated factors by creating a structural equation model.

**Methods:**

A cross-sectional multistage cluster sampling study was conducted among nursing students within six universities in Shaanxi province, China. A model was constructed, and structured questionnaires were adopted to measure model variables, including e-health literacy, negative emotion, self-efficacy, self-care agency, degree of dysmenorrhea, and self-care behavior for dysmenorrhea. Descriptive data analysis was performed using SPSS 23.0 software, and AMOS 23.0 was used to verify and analyze the structural model.

**Results:**

In total, 1851 valid questionnaires were collected; the effective recovery rate was 93.15%, and the prevalence of dysmenorrhea was 64.51%. e-Health literacy (*B* = 0.171, *P* < 0.001), self-efficacy (*B* = 0.416, *P* < 0.001), self-care agency (*B* = 1.177, *P* < 0.001), and degree of dysmenorrhea (*B* = 0.310, *P* < 0.001) significantly influenced self-care behavior for dysmenorrhea. The total, direct, and indirect effects of e-health literacy and self-efficacy on self-care behavior for dysmenorrhea were 0.158 and 0.492, 0.128 and 0.248, and 0.030 and 0.244, respectively.

**Conclusion:**

The self-care behavior for dysmenorrhea is affected by several factors and self-efficacy has the greatest effect on it. To promote girls to actively implement self-care behavior for dysmenorrhea, educators should strengthen the training of self-efficacy and self-care agency of the nursing students to alleviate the uncomfortable experience brought by dysmenorrhea and decrease the harm of dysmenorrhea. *Implications for Nursing Management*. Nursing managers should work with constant efforts to explore and optimize the management model for dysmenorrhea, encouraging young women to actively engage in self-care behavior for dysmenorrhea, to alleviate the discomfort experienced by individuals and improve women's overall health.

## 1. Background

Dysmenorrhea is defined as recurrent spasmodic pain in the lower abdomen during menstruation and is one of the most common causes of pelvic pain and menstrual disorders [1]. According to relevant reports, 56.4%–90% of women have dysmenorrhea [2–4], which is the main cause of their absenteeism from school and work [5, 6]. Dysmenorrhea is clinically divided into primary and secondary dysmenorrhea [7]. From the viewpoint of individual physical and mental development, severe dysmenorrhea can interfere with their daily activities as well as cause varying degrees of harm to their life and quality of life [8, 9], thus adding to a huge public health burden [10]. Conversely, from the social and economic viewpoint, absenteeism caused by dysmenorrhea [11] causes an annual loss of 140 million working hours in the United States and economic losses of over $4.2 billion in Japan, thus making dysmenorrhea one of the important issues that need to be urgently addressed by researchers globally. The level of self-care agency of patients with dysmenorrhea is closely associated with their dysmenorrhea status [12]. Therefore, one of the most important approaches for alleviating dysmenorrhea symptoms and reducing the harm of dysmenorrhea is whether individuals can fully take the initiative and be enthusiastic to implement self-care behaviors beneficial for their health.

According to the latest revision of the WHO Guidelines on self-care interventions [13], self-care is defined as the ability of individuals, families, and communities to promote health, prevent disease, maintain health, and cope with illness and disability, with or without the support of health workers. As a new primary healthcare approach [14], self-care has the potential to save health system resources and reduce patient care and follow-up costs [15, 16]; furthermore, it is cost-effective and easy to learn [17]. Self-care has been widely used in postpartum women, those with spinal cord injury, those who underwent cancer surgery, and those with chronic diseases and plays an important role in the clinical outcome and prognosis of patients with chronic diseases [18–25]. Meanwhile, nursing students, as a special group, have been shown that the implementation of self-care behavior can not only help nursing students relieve pressure and make them better transition to nurse status but also help nurses and patients jointly create a safe clinical environment [26]. However, there are few research studies on the combination of nursing students' dysmenorrhea and self-care behavior as the research direction, the existing research studies are mostly single-factor or cross-sectional investigations, and the holistic research based on the scientific nursing theory is relatively lacking. Therefore, in this study, taking Orem's self-care theory as the theoretical framework, a structural equation model was created to conduct a multidimensional investigation and analysis of the self-care behavior of nursing students, discussed the status quo of nursing students self-care behavior for dysmenorrhea and related influencing factors, and provided reference and basis for medical personnel to frame effective intervention measures and guide these nursing students in actively implementing self-care behavior.

### 1.1. Literature Review

Dysmenorrhea is one of the most common gynecological disorders affecting the quality of life and social activities of women [27]. However, only 20.8% of the students choose to seek medical help for alleviating the discomfort and pain caused by dysmenorrhea; the vast majority of students prefer self-care [28]. The self-care behavior for dysmenorrhea is affected by many factors, among which self-care agency has the greatest impact on it, demonstrating a positive correlation and direct effect [29, 30]. Wong et al. [30] pointed out that previous knowledge regarding menstruation has direct and indirect effects on self-care behaviors for dysmenorrhea and that there is a direct effect between the mother's education level and degree of dysmenorrhea and self-care behaviors for dysmenorrhea. The higher the e-health literacy [31, 32] and the better the individuals' self-efficacy [33, 34], the more willing they are to adopt self-care behaviors that can improve their health status and alleviate their pain. However, thus far, the existing studies on self-care for dysmenorrhea are mostly single factor, and there is a lack of systematic research on the relationship between multiple variables based on scientific theories and models. Therefore, to help medical staff identify the potential or existing wrong self-care behavior of patients with dysmenorrhea as early as possible and improve self-care agency, it is important to assess the self-care behavior of these patients.

### 1.2. Structural Equation Model

This study is guided by Orem's self-care theory, which considers that each individual can independently implement self-care behavior [35] and that nursing intervention aims to help individuals improve their self-care agency [36].

Orem's self-care theory comprises self-care, self-care defect, and nursing system theories [37]. The self-care theory can provide the foundation for explaining self-care behavior, assessing the relationship between relevant variables, and predicting the effect of self-care intervention. This theory points out that self-care agency is an important requirement for individuals to implement self-care behavior and that its strength is closely associated with basic condition factors (BCFs). BCFs include age, sex, developmental status, health status, sociocultural context, health reasons (e.g., medical diagnoses and therapeutic interventions), family factors, lifestyle, environmental factors, and availability [38]. All BCFs can affect the level of self-care agency and are used in self-care behavior, that is, BCFs affect self-care behavior through self-care agency [37].

Resource availability and its adequacy are one of the BCFs that have a direct impact on self-care agency [37]. e-Health literacy as a reflection of the ability to acquire and apply network resources demonstrates that [39, 40] the level of e-health literacy of individuals directly affects their ability to care for poor health. The pain intensity during dysmenorrhea is a distress signal released by the body after an individual's health condition is compromised; the pain degree of women with dysmenorrhea is significantly associated with their self-care behavior [30, 41]. Therefore, in the model framework of the present study, from Orem's self-care theory, resource availability and the health status were selected as exogenous variables and self-care agency and self-care behavior as media and outcome variables, respectively.

**Figure 1:**
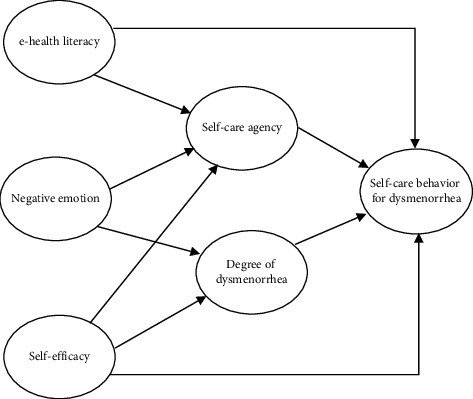
Theoretical framework.

Furthermore, the path relationship between the factors was established based on the findings from the existing research. Negative emotional experiences can decrease the pain threshold and increase the pain degree of individuals, leading to a vicious cycle of negative emotional responses to dysmenorrhea [42]; nonetheless, negative emotions have a significant negative relationship with self-care agency [43, 44]. Individuals with high self-efficacy are frequently able to actively learn relevant knowledge, take correct and positive nursing measures, improve their self-care agency, and alleviate their pain and discomfort through continuous learning [45]. These individuals also display higher self-care abilities and implement self-care behaviors that are favorable to symptom relief or improvement of their condition [46]. Although Orem's self-care theory does not highlight the association among negative emotion, self-efficacy, and degree of dysmenorrhea, previous studies have confirmed the significant association between the abovementioned variables; therefore, the path generated by the abovementioned variables was included in the present model. To sum up, the following hypothesis model was proposed after integrating Orem's self-care theory with the findings of previous studies (Figure 1).

### 1.3. Purpose

This study aimed to create a structural equation model and use it to clarify the influencing factors and the relationship between the variables of the self-care behavior of nursing students with dysmenorrhea in China to provide a theoretical basis and reference for medical staff and educators to better provide dysmenorrhea-related diagnosis and develop treatment strategies and scientific protective behaviors.

## 2. Methods

### 2.1. Overview

In this study, a cross-sectional multistage cluster sampling survey was conducted among nursing students from six universities in Shaanxi province, China. The structural equation model was used to assess the relationship between various variables and self-care behaviors for dysmenorrhea.

### 2.2. Participants and Data Collection

From February 2023 to April 2023, a multistage cluster sampling method was used for recruiting students. First, Shaanxi province in northwest China was chosen as the sampling unit among the seven major geographical regions in China. Second, according to the size and category of the university, six medical colleges were chosen from 15 universities offering undergraduate nursing majors in Shaanxi province and using the convenience sampling method to select eight classes in first- and second-year nursing undergraduate students from each medical college, resulting in a total of 48 classes. Finally, all nursing students from the selected classes who met the inclusion criteria were taken as subjects.

Inclusion criteria were as follows: (1) consistent with the diagnosis of dysmenorrhea [47] and (2) not having a psychiatric illness. Exclusion criteria included communication difficulties, poor compliance, and inability to complete the questionnaire.

According to the calculation method of the sample size of the structural equation, the baseline sample size was 15–20 times the predictive variables [48, 49] and the number of measured variables in this study was 23. By calculating 20 times the measured variables, 460 subjects should have been selected. In this study, it was calculated by the following equation: (3 + 3+4 + 4+3 + 6) *∗* 20 = 460. A total of 1,987 questionnaires were sent to six schools in this study, and 1,851 valid questionnaires were finally recovered after excluding those that did not meet the inclusion criteria. The effective recovery was 93.15%.

### 2.3. Measurement Tools

#### 2.3.1. e-Health Literacy Scale

The e-Health Literacy Scale [50], which includes eight items and wherein each item adopts the Likert 5-level scoring method, including three dimensions, application ability, evaluation ability, and decision-making ability of online health information and services, was used as an assessment tool. The Cronbach's *α* coefficient of the Chinese version of the scale was 0.913 [51], and Cronbach's *α* was 0.960 in this study.

#### 2.3.2. Depression Anxiety Stress Scale-Simplified Version

A simplified version of the Depression Anxiety Stress Scale [52], which includes depression, anxiety, and stress with a total of 21 items, was used to measure the negative emotions of the study subjects. The Likert 5-level scoring method was used. In this study, the Cronbach's *α* coefficient of the Chinese version of the scale was 0.890 [53] and Cronbach's *α* was 0.969.

#### 2.3.3. Self-Rated Abilities for the Health Practices Scale

The Self-Rated Abilities for Health Practices Scale compiled by Becker et al. was used to measure self-efficacy [54]. This tool was translated from English into Chinese with a total of 28 items and four dimensions that represented the ability to promote healthy behavior in four aspects, namely, nutrition, exercise, psychological well-being, and health responsibility. The Likert 5-level scoring method was used. In this study, the Cronbach's *α* coefficient of the Chinese version of the scale was 0.950 [55] and Cronbach's *α* was 0.976.

#### 2.3.4. Self-Care Agency Scale

The Exercise of Self-Care Agency Scale translated into Chinese by Kearney and Fleischer and [56] and Taiwan scholars [57] as a research tool with a total of 43 items and four dimensions, including the health knowledge level, self-concept, self-care responsibility, and self-care skills, was used. The Likert 5-level scoring method was used. In this study, the Cronbach's *α* coefficient of the Chinese version of the scale was 0.890–0.920 [57] and Cronbach's *α* was 0.931.

#### 2.3.5. Degree of Dysmenorrhea Scale

The Menstrual Distress Questionnaire, developed by Moos [58], which includes two subscales of physical dysmenorrhea symptoms and mental and psychological symptoms before menstruation, each with three dimensions and a total of 30 items, was used. The subscale of physical dysmenorrhea symptoms in the Menstrual Distress Questionnaire, including pain-related symptoms, autonomic nervous disorder symptoms, and water and sodium retention symptoms, with a total of 14 items, was selected. The Likert 5-level scoring method was used. The higher the score, the more severe the dysmenorrhea. In this study, the Cronbach's *α* coefficient of the Chinese version of the scale was 0.937 [59] and Cronbach's *α* was 0.943.

#### 2.3.6. Adolescent Dysmenorrhea Self-Care Scale

The Adolescent Dysmenorrhea Self-Care Scale developed and compiled by Hsing et al. [60], and translated into Chinese and Cantonese by Wong et al. [61] (Hong Kong, China), is a research tool for measuring self-care behavior of nursing students with dysmenorrhea. The scale includes six dimensions of knowledge acquisition, emotional expression, seeking help, control of external factors, resource utilization, and self-control, with a total of 35 items. The Likert 6-level scoring method was used. In this study, the Cronbach's *α* coefficient of the Chinese version of the scale was 0.940 [61] and Cronbach's *α* was 0.973.

### 2.4. Data Analysis

The data collected in this study were directly exported from the questionnaire platform. IBM SPSS Statistics version 23 software and IBM SPSS AMOS version 23 software were used to analyze. The measurement data were represented by ($\bar{x}\pm s$); the counting data were statistically described by frequency and probability. The structural equation model was composed of two major parts as follows: the measurement model and the structural model. In the measurement model, confirmatory factor analysis (CFA) and the Pearson correlation coefficient were used to test the reliability and validity. In the structural model, the comparative fit index (CFI > 0.90), normed fit index (NFI > 0.90), Tucker–Lewis index (TLI > 0.90), and root mean square error of approximation (RMSEA < 0.06) were analyzed for model fit [62–64]. Bootstrapping analysis was used for the indirect effects, total effects, and statistical significance of the model, and the bootstrap ML method was utilized; the results of this method are more stable [65] and more accurate than other methods in studies with a sample size greater than 200 [66]. The 95% bias-corrected confidence intervals were used and the number of repeated samples was 5000 times. Two-sided *P* values less than 0.05 were considered significant.

### 2.5. Ethical Considerations

This study has been approved by the Ethics Committee of the Xi'an Medical University (number: XYYJSLS2022067). The participants voluntarily enrolled in this investigation and provided signed informed consent before the study.

## 3. Results

### 3.1. Characteristics

The number of valid questionnaires in this study was 1,871, of which 1207 cases (64.51%) had dysmenorrhea. The average age of the subjects was 19.97 ± 1.27 years and approximately half of the girls had menarche at the age of 13–14 years (49.3%). Among the nursing students with dysmenorrhea, 186 (15.4%) had very irregular menstrual periods and the average number of menstrual days was 5–7 days in 826 (67.6%). Other general information is shown in Table 1.

### 3.2. Measurement Model

First, the original measurement model was verified, and the measurement variable with a standard factor load value below 0.7 was deleted [67, 68], that is, the self-control dimension of dysmenorrhea self-care behavior, resulting in a final measurement model comprising six latent variables and twenty-two measured variables.

Second, confirmatory factor analysis was conducted on the final measurement model. The results indicated that all factors had an average variance extracted (AVE) greater than 0.5 and construct reliability (C.R) higher than 0.7 [69, 70], demonstrating good convergent validity of the data in this study (Table 2).

Finally, based on Pearson correlation analysis, the self-care agency for dysmenorrhea was significantly positively correlated with e-health literacy, self-efficacy, self-care agency, and degree of dysmenorrhea (*r* = 0.318, *P* < 0.001; *r* = 0.503, *P* < 0.001; *r* = 0.524, *P* < 0.001; *r* = 0.058, *P* < 0.05) and negatively correlated with negative emotion (*r* = −0.098, *P* < 0.01) (Table 3). The results of the discriminant validity test in the measurement model demonstrated that the correlation coefficients between variables were all lower than the square root of AVE, indicating good discriminant validity of the model (Table 3).

### 3.3. Structural Equation Model

**Figure 2:**
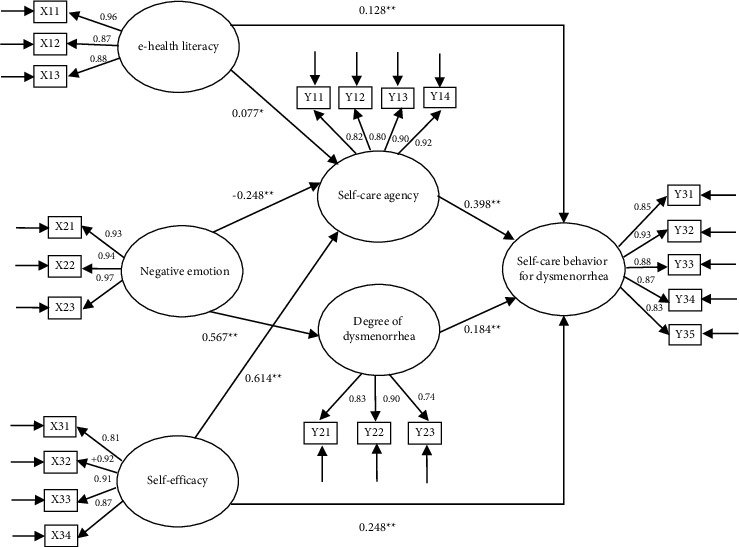
Structural equation model of self-care behavior of medical students with dysmenorrhea. *X*11: test the application capability of network health information and service; *X*12: judging ability; *X*13: decision-making ability; *X*21: depressed; *X*22: anxiety; *X*23: stress; *X*31: nourishment; *X*32: movement; *X*33: psychological comfort; *X*34: health responsibility; *Y*11: health knowledge level; *Y*12: self-concept; *Y*13: self-care responsibility; *Y*14: self-care skills; *Y*21: pain-related symptom; *Y*22: autonomic nervous disorder; *Y*23: water and sodium retention; *Y*31: acquired knowledge; *Y*32: emotional expression; *Y*33: ask for help; *Y*34: control external factors; *Y*35: resource utilization. ^*∗*^*P* < 0.05, ^*∗∗*^*P* < 0.01.

The theoretical framework (Figure 1) drew a total of nine paths; the hypothetical model is constructed based on the framework, and the result of the model fit showed that RMSEA = 0.062, CFI = 0.961, NFI = 0.953, and TLI = 0.954. According to the results of the software correction index, the model was further modified, the path between self-efficacy and dysmenorrhea in the hypothetical model was removed, and the final model with a total of eight paths was formed and analyzed, as shown in Figure 2. The fit index of the modified model showed the following: RMSEA = 0.058, CFI = 0.967, NFI = 0.959, and TLI = 0.962.

The parameter estimation table of the modified model for the self-care behavior of nursing students with dysmenorrhea is shown in Table 4. This study showed that eight out of nine pathways were statistically significant, whereas the path from self-efficacy to dysmenorrhea showed no statistical significance. Among them, the explanation rate of the degree of self-care behavior explained by self-care agency, degree of dysmenorrhea, e-health literacy, and self-efficacy was 41.7%, that of self-care agency explained by e-health literacy, negative emotions, and self-efficacy was 53.7%, and that of the degree of dysmenorrhea explained by negative emotions was 32.1%.

### 3.4. Effects

In the present study, self-care agency had the greatest effect on the outcome variable self-care behavior for dysmenorrhea and self-efficacy had the greatest effect on the media variable self-care agency. The direct and overall effects of the eight pathways and the indirect effects of e-health literacy and self-efficacy on self-care behavior for dysmenorrhea were statistically significant (Table 5).

## 4. Discussion

### 4.1. Current Situation of the Self-Care Behavior of Nursing Students with Dysmenorrhea

According to the general characteristics of the population included in this study, approximately half of the girls had menarche at the age of 13–14 years (49.3%) and the average number of menstrual days was 5–7 days in 826 (68.4%). The incidence of dysmenorrhea was about 64.51% (1207/1871), which was similar to the findings of a previous study [71], indicating that dysmenorrhea is still a major problem that should be paid more attention to during the healthy development of female body and mind. Most individuals with dysmenorrhea have menarche at the age of 13-14 years, accounting for 49.5%. It is suggested that schools should form a relationship education mechanism with local primary healthcare departments and parents of students and conduct activities including lectures or exchange forums related to menstruation to establish correct cognition of normal physiological phenomena such as menstruation in individuals and encourage them to take positive and correct self-care behaviors for dysmenorrhea spontaneously, thus helping them alleviate the discomfort caused by dysmenorrhea.

### 4.2. Fit Degree of the Structural Equation Model for the Self-Care Behavior of Nursing Students with Dysmenorrhea

This study took Orem's self-care theory as the core framework. After the verification and modification of the structural model, the fit degree of the structural equation model for the self-care behavior of nursing students with dysmenorrhea was at a good level. Self-care agency, degree of dysmenorrhea, e-health literacy, self-efficacy, and other independent variables directly affected the self-care behavior for dysmenorrhea, and the explanation rate of the abovementioned variables was 41.7%. Among them, e-health literacy, self-efficacy, and self-care behavior for dysmenorrhea had indirect effects. The variables that directly affected self-care agency were e-health literacy, negative emotion, and self-efficacy; the explanation rate of these variables was 53.7%. Furthermore, negative emotion was a factor that directly affected the degree of dysmenorrhea; the explanation rate of this factor was 32.1%.

### 4.3. Mediating Effect of Various Variables on the Self-Care Behavior of Nursing Students with Dysmenorrhea

Individual e-health literacy can not only directly affect the self-care behavior for dysmenorrhea but also indirectly affect self-care agency. Similarly, self-efficacy not only has a direct effect on the self-care behavior for dysmenorrhea but also indirectly affects the self-care behavior of individuals through self-care agency. This fully demonstrates that the self-care agency plays a critical role in e-health literacy, self-efficacy, and self-care behavior for dysmenorrhea. Therefore, healthcare departments or universities should be fully aware of self-care agency when conducting menses-related education for nursing students and boost self-care agency by improving individual e-health literacy and guiding students to improve self-efficacy to achieve the aim of actively implementing self-care behaviors for dysmenorrhea.

### 4.4. Limitations

This study has two limitations. First, the sample size is confined to nursing students exclusively from select medical colleges in Shaanxi province, China, which may restrict the generalizability and diversity of the findings. To enhance the universality and persuasiveness of the research results, it is recommended to expand the types and range of samples in future studies. Second, due to the inherent constraints of cross-sectional research regarding time relationships and causality, establishing associations between variables in this study relies on theoretical frameworks and previous literature. Therefore, conducting longitudinal or interventional studies on factors influencing self-care behaviors for dysmenorrhea among nursing students would be advantageous for further exploration of predictive factors impacting such behaviors.

## 5. Conclusion

Based on Orem's self-care theory and the findings of previous studies, this study created a scientific structural equation model that can explain the factors affecting self-care behavior for dysmenorrhea. The model verified and analyzed the explanation degree of e-health literacy, negative emotion, and self-efficacy on self-care behavior for dysmenorrhea, and the mediating role and effect size of the self-care agency and degree of dysmenorrhea.

## 6. Implications for Nursing Management

Dysmenorrhea is a significant factor affecting women's health. It is not merely a temporary issue for individuals, schools, or families but also a major concern related to students' academic performance and future fertility. It profoundly impacts their overall well-being, future life, and work. Implementing proactive and effective self-care behaviors can alleviate the discomfort and enhance their quality of learning and life. Schools and families should offer proper guidance to students with dysmenorrhea, foster the development of correct cognitive attitudes, and establish positive coping strategies. Nursing managers can refine the management model for self-care behaviors associated with dysmenorrhea. With the advancements in the Internet and big data, they can create authoritative and scientific information platforms for students to access and reference, thereby reducing the pressure on students' e-health literacy. In addition, considering individual personality traits and lifestyle habits, tailored self-care recommendations can be provided, thereby reducing menstrual discomfort and furthering women's health.

## Figures and Tables

**Table 1:** General characteristics.

Factors	Groups	No dysmenorrhea	Frequency (%)	$\bar{x}\pm s$	Dysmenorrhea	Frequency (%)	$\bar{x}\pm s$
Age	≤18	77	4.2	19.97 ± 1.27	65	5.4	20.07 ± 1.29
	19–21	1546	83.5		992	82.2	
	≥22	228	12.3		150	12.4	

BMI (kg/m^2^)	Emaciation (<18.5)	483	26.1	20.39 ± 2.83	305	25.3	20.60 ± 3.20
	Normal (18.5 ≤ BMI < 24)	1171	63.3		767	63.5	
	Overweight (24 ≤ BMI < 28)	169	9.1		98	8.1	
	Obesity (≥28)	28	1.5		37	3.1	

Age of menarche	≤12	643	34.7	13.18 ± 1.36	431	35.7	13.15 ± 1.37
	13–14	913	49.3		597	49.5	
	≥15	295	15.9		179	14.8	

Menstrual cycle	Very regular	715	38.6		434	36.0	
	Relatively regular	867	46.8		587	48.6	
	Very irregular	269	14.5		186	15.4	

Menstrual period	2–4 days	549	29.7		346	28.7	
	5–7 days	1252	67.6		826	68.4	
	>7 days	50	2.7		35	2.9	

Total		1,851			1,207		

**Table 2:** Verification results of confirmatory factor analysis.

Latent variable	Item	Factor load (FL)	Average variance extracted (AVE)	Construct reliability (C.R)
e-Health literacy	*X*11	0.962	0.864	0.950
	*X*12	0.872		
	*X*13	0.884		

Negative emotion	*X*21	0.926	0.948	0.982
	*X*22	0.943		
	*X*23	0.966		

Self-efficacy	*X*31	0.810	0.830	0.951
	*X*32	0.917		
	*X*33	0.907		
	*X*34	0.867		

Self-care agency	*Y*11	0.824	0.924	0.980
	*Y*12	0.802		
	*Y*13	0.899		
	*Y*14	0.919		

Degree of dysmenorrhea	*Y*21	0.832	0.745	0.897
	*Y*22	0.901		
	*Y*23	0.735		

Self-care behavior for dysmenorrhea	*Y*31	0.853	0.694	0.919
	*Y*32	0.927		
	*Y*33	0.881		
	*Y*34	0.865		
	*Y*35	0.824		

*X*11: test the application capability of network health information and service; *X*12: judging ability; *X*13: decision-making ability; *X*21: depressed; *X*22: anxiety; *X*23: stress; *X*31: nourishment; *X*32: movement; *X*33: psychological comfort; *X*34: health responsibility; *Y*11: health knowledge level; *Y*12: self-concept; *Y*13: self-care responsibility; *Y*14: self-care skills; *Y*21: pain-related symptom; *Y*22: autonomic nervous disorder; *Y*23: water and sodium retention; *Y*31: acquire knowledge; *Y*32: emotional expression; *Y*33: ask for help; *Y*34: control external factors; *Y*35: resource utilization.

**Table 3:** Average scores of each variable item, discriminant validity, and Pearson correlation coefficient (*n* = 1207; SD, standard deviation).

	Mean (SD)	Range	*X*1	*X*2	*X*3	*Y*1	Y2	
e-Health literacy (*X*1)	3.91 [0.79]	1.00–5.00	**0.930**					
Negative emotion (*X*2)	1.49 [1.31]	0.00–6.00	−0.099^*∗∗*^	**0.974**				
Self-efficacy (*X*3)	2.17 [0.75]	0.04–4.00	0.343^*∗∗∗*^	−0.166^*∗∗∗*^	**0.911**			
Self-care agency (*Y*1)	2.40 [0.47]	1.12–4.00	0.314^*∗∗∗*^	−0.351^*∗∗∗*^	0.650^*∗∗∗*^	**0.962**		
Degree of dysmenorrhea (*Y*2)	2.44 [0.76]	1.00–5.00	−0.092^*∗∗*^	0.519^*∗∗∗*^	−0.123^*∗∗∗*^	−0.195^*∗∗∗*^	**0.863**	
Self-care behavior for dysmenorrhea (*Y*3)	3.74 [0.99]	1.09–6.00	0.318^*∗∗∗*^	−0.098^*∗∗*^	0.503^*∗∗∗*^	0.524^*∗∗∗*^	0.058^*∗*^	**0.833**

Diagonal bold numbers are AVE square root values, and numbers below diagonal are Pearson correlation coefficients (^*∗*^*P* < 0.05, ^*∗∗*^*P* < 0.01, ^*∗∗∗*^*P* < 0.001). If the AVE square root value is greater than the correlation coefficients of the corresponding variable, it indicates good discriminant validity of the measured variable.

**Table 4:** Parameter estimates of variables for the modified model.

Paths	*B* (S.E.)	*β*	*P* value	SMC
Self-care agency: self-care behavior for dysmenorrhea	1.177 (0.111)	0.398	<0.001	0.417
Degree of dysmenorrhea: self-care behavior for dysmenorrhea	0.310 (0.044)	0.184	<0.001	
e-health literacy: self-care behavior for dysmenorrhea	0.171 (0.035)	0.128	<0.001	
Self-efficacy: self-care behavior for dysmenorrhea	0.416 (0.061)	0.248	<0.001	

e-health literacy: self-care agency	0.035 (0.011)	0.077	0.001	0.537
Negative emotion: self-care agency	−0.146 (0.014)	−0.248	<0.001	
Self-efficacy: self-care agency	0.348 (0.017)	0.614	<0.001	

Negative emotion: degree of dysmenorrhea	0.585 (0.030)	0.567	<0.001	0.321

S.E.: standard error, *β*: standardized estimate, SMC: squared multiple correlation.

**Table 5:** Standardized direct, indirect, and total effects.

Dependent variable	Independent variable	Direct effect	Indirect effect	Total effect
Self-care behavior for dysmenorrhea	Self-care agency	0.398^*∗∗*^	—	0.398^*∗∗*^
	Degree of dysmenorrhea	0.184^*∗∗*^	—	0.184^*∗∗*^
	e-Health literacy	0.128^*∗∗*^	0.030^*∗*^	0.158^*∗∗*^
	Self-efficacy	0.248^*∗∗*^	0.244^*∗∗*^	0.492^*∗∗*^

Self-care agency	e-Health literacy	0.077^*∗*^	—	0.077^*∗*^
	Negative emotion	−0.248^*∗∗*^	—	−0.248^*∗∗*^
	Self-efficacy	0.614^*∗∗*^	—	0.614^*∗∗*^

Degree of dysmenorrhea	Negative emotion	0.567^*∗∗*^	—	0.567^*∗∗*^

^*∗*^ *P* < 0.01, ^*∗∗*^*P* < 0.001.

## Data Availability

The data used to support the findings of this study have not been made available because of person privacy and ethical issues.
